# Clinical Report on the Therapeutic Value of Glyco-Thymoline

**Published:** 1899-03

**Authors:** I. J. Jones

**Affiliations:** 219 E. Sixth Street; Austin, Texas


### Clinical Notes.

### Clinical Report on the Therapeutic Value of Glyco-Thymoline.

BY I. J. JONES, M. D., AUSTIN, TEXAS.

Having had a large experience in the management of old cases of rhinitis, and other chronic nasal and pharyngeal conditions of the aged, I hope that a short report of cases will prove of interest to your readers. At the outset I wish to state that I am not a specialist, and I do not suppose that my experience will be of any value to those who are specialists in these branches, or even to those who have a competent specialist available to whom he can refer his patients. But the general practitioner is daily consulted by people suffering from some of these conditions, and he is often embarrassed for a treatment that is applicable and accessible to himself and his patient.

It was this situation that led me, while acting as surgeon to the Texas Confederate Home, where there are large numbers of these cases, to experiment with Glyco-Thymoline. This is an alkaline, antiseptic liquid, carrying in perfect solution some of the essential oils, and is a local stimulant. Its application with a Bermingham nasal douche (which can be purchased in any drug store for a few cents) is easy, and not at all disagreeable to the patient. The general practitioner can keep a bottle of the preparation and a douche in his office, and at trifling expense be prepared to do *something,* and in very many instances the best thing, for his patients, who would probably otherwise go untreated; and our country brethren, who are distant from the specialsts, could add considerable to his income from this source, and give many times value received for it by adopting this treatment. During the last two and one-half years I have dispensed in my practice about one hundred bottles, to a large variety of patients, but will only report four or five illustrating the range of usefulness of the preparation.

Case I. J. M., æt 65. Male. Atrophic rhinitis of both nares. Turbinates had entirely disappeared. Septum perforated. Odor simply horrible. Large crusts in posterior nares and naso-pharynx. Began treatment with 25 per cent. warm solution Glyco-Thymoline, gradually increased to 50 per cent. administered three times daily. After three weeks treatment the odor had disappeared entirely. No crusts; a frontal headache, which had annoyed him constantly, had now disappeared. He was now directed to keep on hand a douche and supply of the preparation, using it regularly once a day, and oftener if he became worse. Has remained under my daily observation for eighteen months since, and declares that he is perfectly relieved of the symptoms from which he had suffered. Of course the anatomical lesions were not improved.

CASE II. C. J., female, æt 3. Tonsilitis and rhinitis, accompanied by membrane very similar to diphtheria. Putrid odor from nose and mouth. No fever. Had attempted to make a number of applications without much success, owing to the child's resistance. Gave one-tenth grain calomel every hour for ten hours, and applied 25 per cent. Glyco-Thymoline with steam atomizer every three hours. odor disappeared almost at once, and membrane began to loosen and come away in twenty-four hours, and had soon disappeared. Treatment continued at longer intervals for one week, when patient had entirely recovered.

CASE III. C. E. B., æt 60, male. Acute coryza. Frequent sneezing. "Head stopped up," nasal discharge and headache, nasal mucous membrane turgid. Gave atropia sulph. 1-100 gr., and 25 per cent. Glyco-Thymoline hot as could be borne, every two hours, to be applied with douche. Relieved almost immediately.

CASE IV. W. P. H., æt 81. Acute cystitis with acid urine. Urine dark brown and loaded with pus and shreds of mucous membrane, and quantity greatly diminished. Excessive nausea. In addition to appropriate constitutional treatment, the bladder was irrigated with 10 per cent. solution Glyco-Thymoline in warm water, through a Wigmore "Y" three times daily. The condition of the urine was almost immediately improved in a marked manner. Pus disappeared, as also did the mucous shreds. The color soon became normal as well as the reaction, and the quantity was increased. The foul odor, which had characterized the urine at the beginning, was removed by the second irrigation. Owing, however, to the patient's great age, he succumbed to the prostration.

CASE V. J. G., æt 72, male. Large and deep chronic ulceration of leg just above external maleolus. Had persisted for fourteen years. Edges ragged, odor fetid. Cauterized thoroughly with nit. silver stick, washed with 25 per cent. hot solution Glyco-Thymoline. The wash was kept up daily, with one or two more applications of silver nit., for eight months, when the ulcer was entirely healed, and has remained so, under my observation, for eighteen months.

The great beauty of Glyco-Thymoline, like Captain Cuttle's moral, lies in the application of it. It is so simple, feasible, pleasant and effective that I am sure it would become a routine practice with any general practitioner who would give it a trial.

219 E. Sixth Street.

